# A Mechanistic Study of *Lactobacillus helveticus* HY7801 and Its Extracellular Vesicles in Premenstrual Syndrome: Role of Gut Microbiota and Hormonal Modulation

**DOI:** 10.4014/jmb.2507.07014

**Published:** 2025-09-11

**Authors:** Hyeonji Kim, Hyeonjun Gwon, Ji-Woong Jeong, Joo-Yun Kim, Jae-Jung Shim, Jae-Hwan Lee

**Affiliations:** R & BD Center, hy Co. Ltd., Yongin-si 17086, Republic of Korea

**Keywords:** Premenstrual syndrome (PMS), probiotics, Gut microbiota, extracellular vesicle (EV), prolactin

## Abstract

Premenstrual syndrome (PMS) is characterized by periodic physical and psychological symptoms during the menstrual cycle of women of reproductive age. This study aimed to investigate the effects of *Lactobacillus helveticus* HY7801 (HY7801) on PMS-related hormonal and inflammatory responses, with a specific focus on its influence on the composition of the gut microbiota and the functional role of HY7801-derived extracellular vesicles (EVs). Data from a metoclopramide (MCP)-induced hyperprolactinemia mouse model showed that oral administration of HY7801 reduced serum levels of prolactin and pro-inflammatory cytokines significantly, while restoring hormone imbalances caused by MCP. HY7801 also altered the composition of the gut microbiota by decreasing the relative abundance of *Desulfovibrionaceae*, *Staphylococcaceae*, and *Bacteroidaceae*, which are bacterial families associated with mental health disorders. The abundance of these taxa correlated positively with prolactin and cytokine levels. Furthermore, HY7801-derived EVs were identified as functional bioactive components that exert cytoprotective effects and suppress prolactin secretion significantly in estradiol-stimulated GH3 cells. These findings indicate that HY7801 alleviates PMS symptoms, not only by modulating gut microbiota but also through direct cellular mechanisms mediated by its EVs. Taken together, these dual mechanisms suggest that HY7801 and its secreted components represent a promising approach to management of PMS symptoms.

## Introduction

Premenstrual syndrome (PMS), which encompasses a wide variety of physical and emotional symptoms that occur 1–2 weeks prior to the onset of menstruation, affects approximately 80–95% of women of reproductive age, with symptom severity ranging from mild to severe [1, 2]. Among those affected by PMS, 2–8% suffer from a more severe form called premenstrual dysphoric disorder [3]. Common symptoms include emotional disturbances such as anxiety, tension, depression, and mood swings, as well as physical discomfort, including headaches, fatigue, abdominal bloating, and breast tenderness [4]. These symptoms can impair personal, professional, and social functioning significantly [5]. Although the exact etiology of PMS remains unclear, various contributing factors have been proposed, including increased secretion of, or sensitivity to, prolactin, fluctuations in hormones (*e.g.*, estrogen and progesterone), imbalances in neurotransmitters (*e.g.*, serotonin and gamma-aminobutyric acid), dietary deficiencies, and genetic predisposition [6].

Prolactin, a hormone secreted by the pituitary gland, is involved in secretion of breast milk [7]. Although prolactin levels can fluctuate during the menstrual cycle, abnormally high secretion interferes with the normal production of other hormones such as estrogen, progesterone, and follicle-stimulating hormones (FSH), particularly in women [8, 9]. In addition, elevated prolactin levels during the luteal phase may play a critical role in causing some symptoms of PMS, including premenstrual breast pain, metabolic alterations, and abnormal immune responses [6].

Extracellular vesicles (EVs) are nanosized vesicles enclosed by a phospholipid bilayer, secreted by various organisms including bacteria, eukaryotic cells, and archaea. Typically, EVs range from 30–200 nm in diameter, and contain bioactive molecules such as proteins, lipids, and nucleic acids [10, 11]. Microbial-derived EVs have emerged as crucial mediators of host-microbe communication, and have been shown to modulate host immune responses [12]. Recent research has demonstrated that extracellular vesicles (EVs) derived from probiotic bacteria can modulate host physiology through both local interactions in the intestinal tract and systemic pathways. These vesicles contribute to maintaining mucosal barrier integrity, regulating inflammatory responses, and supporting dermatological health and metabolic balance. In particular, EVs from *Lactobacillus* strains have been shown to enhance microbial composition, reinforce epithelial defenses, suppress inflammation, and facilitate skin regeneration and anti-aging processes [12-14].

In 2001 the FAO/WHO defined probiotics as “Live microorganisms that when administrated in adequate amounts confer a health benefit on the host” [15]. Recent studies reported that probiotics exert a variety of host beneficial effects on the gastrointestinal tract and gut flora balance, as well as mental health (*e.g.*, depression and anxiety) [15-17]. *Lactobacillus helveticus* (*L. helveticus*) is a probiotic strain that has various potential health-improving effects, including overall gut health, immune function, liver injury alleviation, skin barrier function, and vaginal health [18-20]. In our previous study, we demonstrated that *L. helveticus* HY7801 (HY7801) alleviates PMS symptoms in a metoclopramide (MCP)-induced mouse model by reducing prolactin levels, regulating prostaglandin E1/E2, and recovering endometrium thickness [21]. However, despite evidence that probiotic strain HY7801 ameliorates the symptoms of PMS, its effect on restoring alterations in the gut microbiota caused by metoclopramide (MCP) have not yet been studied. In addition, it is unclear which bioactive components derived from HY7801 support the mechanism that improves PMS symptoms.

The aim of this study was to identify the mechanisms by which *L. helveticus* HY7801 alleviates the symptoms of PMS. Specifically, we used a hyperprolactinemia-induced mouse model to investigate whether HY7801 modulates PMS-related biomarkers via alterations in the gut microbiota. In parallel, we explored whether EVs, secreted by HY7801, regulate prolactin production directly in estradiol-stimulated GH3 pituitary cells, thereby acting as bioactive components mediating its therapeutic effects.

## Materials and Methods

### Bacteria Culture and Sample Preparation

*L. helveticus* HY7801 (HY7801) was cultured in MRS broth (BD Difco, USA) at 37°C for 24 h. The freshly grown cells were lyophilized and incorporated into the feed for *in vivo* experiments. Extracellular vesicles were obtained from the culture supernatant using an ExoTFFTM Kit (Microgentas, Republic of Korea), suspended in sterile PBS, and stored at −80°C until further use in cell-based assays.

### Animal Experiments

Female ICR mice (14 weeks old) were purchased from Central Lab Animal Inc. (Republic of Korea) and maintained for 1 week under controlled conditions (50 ± 10% humidity at 23 ± 2°C, with a 12 h light/dark cycle). After adaptation, mice were assigned randomly into one of the following four groups (*n* = 9/group): A non-treatment group (Control); a metoclopramide-induced group (MCP, 20 mg/kg/day); an MCP-induced prefemin (100 mg/kg/day)-administered group; and MCP-induced *L. helveticus* HY7801 (10^9^ colony-forming units [CFU]/kg/day)-administrated group (HY7801). All mice (except the Control group) were injected intraperitoneally with MCP once every 2 days during the experiments (21 days). After the experiments, mice were sacrificed by carbon dioxide (CO_2_) inhalation, and blood, uterine, and cecum tissues were collected. All studies were approved by the Institutional Animal Care and Use Committee hy Co., Ltd., Republic of Korea (IACUC approval no. AEC-2024-0003-Y).

### Measurement of Hormones and Pro-Inflammatory Cytokines in Serum

Blood samples were separated to obtain serum and then analyzed at LABISKOMA (Republic of Korea) using a multiplex assay. Levels of serum prolactin, estradiol, progesterone and FSH, as well as tumor necrosis factor-α (TNF) and interleukin-1β (IL-1β), were measured.

### Amplification and Sequencing of the 16S rRNA Gene

Microbial DNA was extracted from cecal tissue samples using the QIAamp Fast DNA Stool Mini Kit (Qiagen, Germany). To analyze the bacterial community structure, the V3–V4 hypervariable regions of the 16S rRNA gene were amplified following the Illumina 16S Metagenomic Sequencing Library Preparation protocol (Illumina, USA). For the initial PCR reaction, 2 ng of genomic DNA was mixed with 5× reaction buffer, 1 mM dNTPs, 500 nM of each primer, and Herculase II Fusion DNA Polymerase (Agilent Technologies, USA). The thermal profile included denaturation at 95°C for 3 min, followed by 25 cycles at 95°C for 30 sec, 55°C for 30 sec, and 72°C for 30 sec, with a final extension step at 72°C for 5 min. The primer set used for amplification was 341F (5'-TCGTCGGCAGCGTCAGATGTGTATAAGAGACAGCCTACGGGNGGCWGCAG-3') and 806R (5'-CTCGTG GGCTCGGAGATGTGTATAAGAGACAGGACTACHVGGGTATCTAATCC-3'). PCR products were purified using AMPure XP magnetic beads (Beckman Coulter, USA). A second round of PCR was performed to incorporate Nextera XT indices, using 2 μl of the purified product as template and the same PCR conditions for 10 cycles. Final library quantification was performed using the KAPA Library Quantification Kit (KAPA Biosystems, USA), and library quality was validated using the Agilent TapeStation D1000 system (Agilent Technologies, Germany). Paired-end sequencing (2 × 300 bp) was carried out on an Illumina MiSeq platform.

### Microbiome Bioinformatic Analysis

Raw sequencing reads were analyzed using QIIME2 (version 2023.9). After demultiplexing, sequences were quality-filtered, denoised, and merged via the DADA2 plugin to generate amplicon sequence variants (ASVs). Taxonomic classification was performed using the QIIME2 feature classifier against the SILVA 138 database (99%identity threshold). Alpha diversity indices (Shannon and Evenness) were calculated with the core-metrics-phylogenetic pipeline, and group differences were tested by the Kruskal–Wallis method. Beta diversity was assessed using weighted UniFrac distances, and Principal Coordinate Analysis (PCoA) was applied for visualization. Statistical significance of community structure was determined by PERMANOVA. Sequencing data are available in the NCBI Sequence Read Archive under accession number PRJNA1277485.

### Correlation Heatmap

A correlation heatmap was constructed using the pheatmap package in the R software (v 4.0.3). The correlation between the relative abundance of intestinal microflora and biochemical indicators was analyzed by calculating Pearson’s coefficient.

### Cell Culture and Sample Treatment

The GH3 epithelial-like cell line was obtained from the American Type Culture Collection (ATCC, USA) and grown at 37°C/5% CO_2_ in Kaighn’s Modification of Ham’s F-12 Medium (Gibco, USA), supplemented with 15%horse serum, 2.5% fetal bovine serum, and 1% penicillin/streptomycin (Gibco). GH3 cells were seeded onto 24-well plates at a density of 1 × 10^5^ cells/well, and stabilized for 24 h. Then, the growth medium was replaced with starvation medium. The wells were pretreated for 4 h with EVs isolated from HY7801 (at concentrations of 0.1, 0.5, 1, 5, 10 μg/ml), followed by addition of 10 nM 17β-estradiol (E2; Sigma-Aldrich, USA) for 18 h.

### Measurement of Prolactin Secreted by GH3 Cells

Prolactin secretion was measured using a Rat PRL/Prolactin ELISA Kit (LS Bio, USA). Absorbance was measured at 450 nm in a BioTek Synergy HT Microplate reader (USA).

### Gene Expression

Total RNA was extracted from uterine tissues and GH3 cells using the Easy-spin RNA Extraction Kit (iNtRON Biotechnology, Republic of Korea). cDNA was synthesized using the Omniscript Reverse Transcription Kit (Qiagen) according to the manufacturer's instructions. Quantitative PCR was conducted using TaqMan^TM^ Gene Expression Master Mix on a QuantStudio 6 Real-Time PCR System (Applied Biosystems). Target-specific probes are listed in Table 1. Gene expression levels were normalized to Gapdh (Mm99999915_g1 for mouse samples, Rn01749022_g1 for GH3 cells).

### Nanoparticle Tracking Analysis (NTA)

NTA was performed with ZETAVIEW (Particle Metrix, Germany) at Microgentas to determine the concentration and size of EVs. The sample conductivity was measured at 15000.00 μS/cm. The instrument parameters used for measurement were a laser wavelength of 488 nm of and a filter wavelength in scattering mode. Samples were measured at a dilution of 1:200. Data were analyzed using ZetaView software (version 8.06.01 SP1).

### Statistical Analyses

The data from the *in vivo* and *in vitro* experiments are presented as the mean ± standard deviation (SD), along with 95% confidence intervals. Data were analyzed by one-way ANOVA with Tukey’s *post-hoc* test using GraphPad Prism 6.0 (USA).

## Results

### Effects of HY7801 on Serum Levels of Sex Hormones

**Fig. 1:**
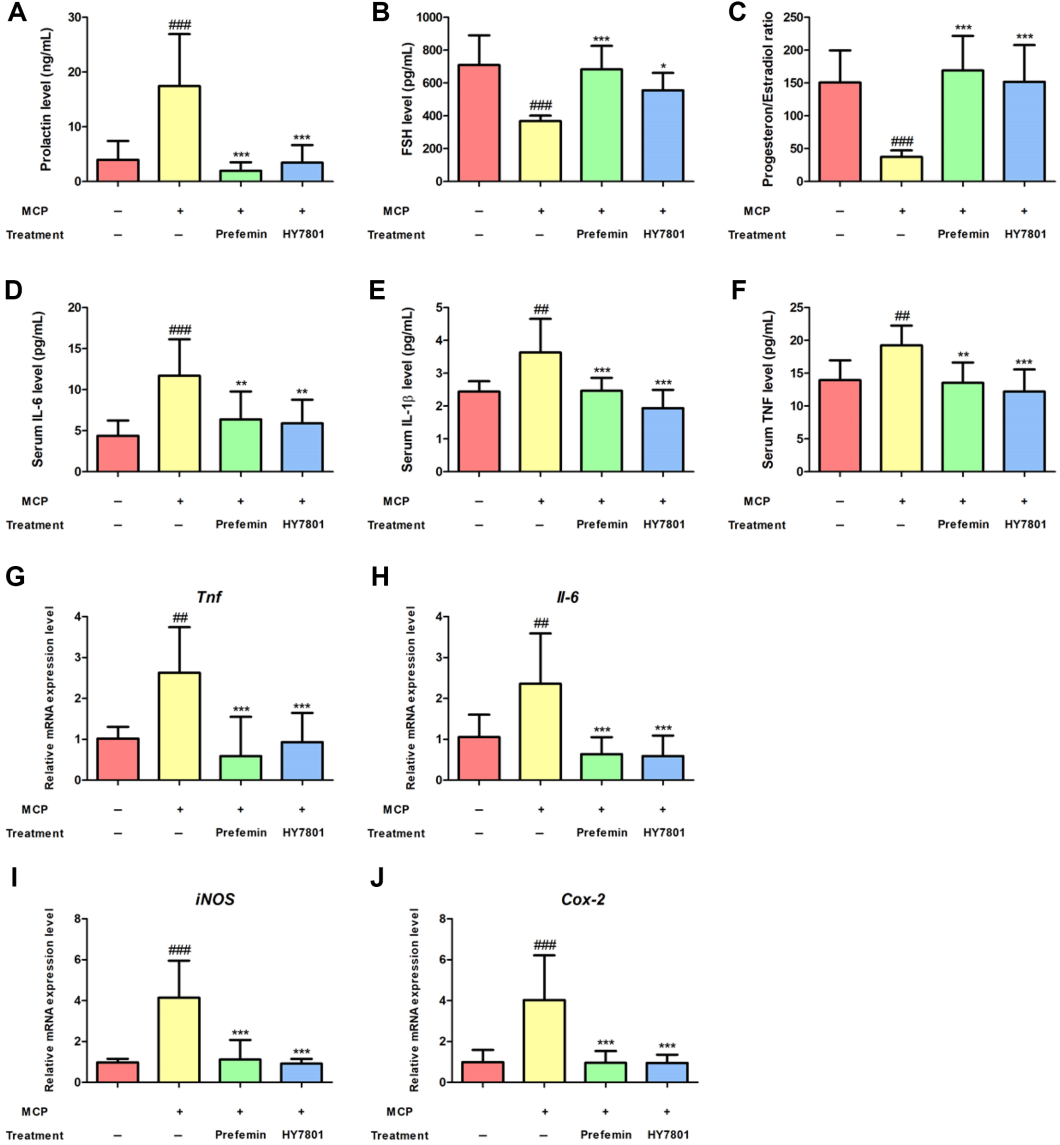
Effect of HY7801 on serum levels of hormones and pro-inflammatory cytokines in in mouse model of hyperprolactinemia. Serum levels of (**A**) prolactin and (**B**) FSH, (**C**) the progesterone/estradiol ratio, and proinflammatory cytokines (**D**) IL-6, (**E**) IL-1β, (**F**) TNF. Relative expression of mRNA encoding (**G**) *Tnf*, (**H**) *Il-6*, (**I**) *iNOS* and (J) *Cox-2* in uterine tissues. Results are expressed as the mean ± standard deviation. *p < 0.05, **p < 0.01 and ***p < 0.001, compared with the MCP group. Control, Non-treatment group; MCP, Metoclopramide-induced group; Prefemin, MCPinduced prefemin (100 mg/kg/day)-administrated group; HY7801, MCP-induced *Lactobacillus helveticus* HY7801 (10^9^ CFU/ kg/day)-administrated group; FSH, follicle-stimulating hormone; IL-6, interleukin-6; IL-1β; interleukin-1 beta; TNF, tumor necrosis factor-alpha, iNOS, inducible nitric oxide synthase; *Cox-2*, cyclooxygenase-2.

To investigate the effects of HY7801 on mice with MCP-induced hyperprolactinemia, we measured the levels of female hormones. Prefemin, the main component of *Vitex agnus-castus* extract, is used widely as a herbal product to relieve symptoms of PMS [22]. The results showed that prolactin levels in the MCP group were significantly higher (by 441%; *p* < 0.01) than those in the Control group (Fig. 1A). Treatment with prefemin and HY7801 treatment led to a significant reduction in prolactin levels to 50% (*p* < 0.001) and 87% (*p* < 0.05), respectively, of that in Control. The FSH level and progesterone/estradiol (P/E) ratio in the MCP group were significantly lower than those in the Control group (by 52% and 24%, respectively; Fig. 1B and 1C); however, levels recovered significantly upon HY7801 administration (to 78% and 99%, respectively, compared with Control group. Prefemin also caused a significant increase in FSH levels and the P/E ratio, to 96% and 116%, respectively, compared with Control.

### Effects of HY7801 on Secretion of Pro-Inflammatory Cytokines

As shown in Fig. 1D-1F, treatment with MCP led to a significant increase in the serum levels of pro-inflammatory cytokines IL-6, IL-1β and TNF (by 267%, 149% and 138%, respectively, compared with the Control group; Fig. 1D–1F). By contrast, administration of HY7801 led to a significantly reduction in levels of IL-6, IL-1β and TNF (by 134%, 79% and 87%, respectively). Similarly, prefemin caused a significant reduction of serum cytokine levels (by 146%, 101% and 97%, respectively).

### Effects of HY7801 on Expression of Genes Encoding Pro-Inflammatory Cytokines

To determine the effects of HY7801 on expression of uterine genes, levels of mRNA encoding genes related pro-inflammatory cytokines were measured (Fig. 1G-1J). The results showed that expression of *Tnf*, *Il-6*, iNOS and *Cox-2* in the MCP group tended to be significantly higher than that in the Control group; however, HY7801 significantly suppressed expression of these genes to levels below those in the MCP group (*Tnf* and *Il-6*, *p* < 0.01; *iNOS* and *Cox-2*, *p* < 0.001). Prefemin reduced expression of cytokines induced by MCP to levels similar to those in HY7801 group. Thus, HY7801 and prefemin exert anti-inflammatory effects in the MCP-induced mouse model, with no significant difference between the two. In other words, HY7801 has the potential to suppress PMS symptoms.

### Effects of HY7801 on the Gut Microbiota of Mice with MCP-Induced Hyperprolactinemia

**Fig. 2:**
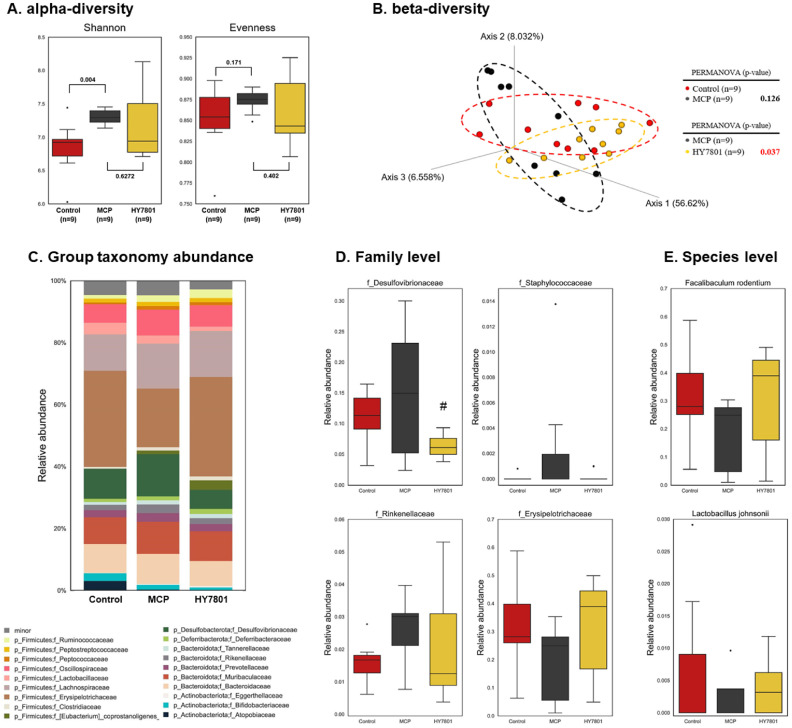
Composition of the gut microbiota in MCP-induced mice. (**A**) alpha diversity, and (**B**) PCoA plot of beta diversity using weighted UniFrac distance. (**C**) Group taxonomy abundance at the family level. Relative abundance at the (**D**) family and (**E**) species level. Red, black, and yellow boxes denote the Control, MCP and HY7801 groups, respectively. Results are expressed as the mean ± standard deviation. ^#^*p* < 0.05, compared with the MCP group. Control, Nontreatment group; MCP, Metoclopramide-induced group; HY7801, MCP-induced *Lactobacillus helveticus* HY7801 (10^9^ CFU/kg/day)-administrated group.

The effects of HY7801 on PMS-induced alterations in the gut microbiota were inferred from metagenomic analysis (Figs. S1 and 2). For α-diversity analysis, the Shannon and Evenness indices were measured. Both of these indices increased in response to MCP, but fell in response to HY7801 (Fig. 2A). By contrast, prefemin resulted in higher α-diversity values than observed for the MCP-induced group (Fig. S1). β-Diversity was examined by analyzing the weighted UniFrac distance to compare the gut microbial composition of each group (Fig. 2B). PCoA revealed that the composition of the gut microbiota in the MCP group was distinct from that of the Control (*p* = 0.013). PcoA separated the prefemin group from the other treatment groups. The HY7801 group was distinct from the MCP group, but the difference was not significant (*p* = 0.060 Next, relative abundance at the family level was investigated (Fig. 2C and 2D). The relative abundance of *Bacteroidaceae*, *Oscillospiaceae*, *Desulfovibrionaceae*, *Staphylococcaceae*, *Prevotellaceae* and *Rikenellaceae* were increased and the relative abundance of *Erysipelotrichiaceae* was decreased in response to MCP. By contrast, there was a tendency for these changes to be reversed by HY7801; alterations in the intestinal microflora caused by MCP was reversed to levels similar to those observed in the Control group. At the species level, an MCP-induced diet reduced the relative abundance of *Faecalibaculum rodentium* (belonging to the *Erysipelotrichiaceae*) and *Lactobacillus johnsonii* compared with the Control group, whereas the relative abundance of these species was increased in the HY7801 group (Fig. 2E).

### Correlation Heatmap between the Gut Microbiota and Indicators of PMS

**Fig. 3:**
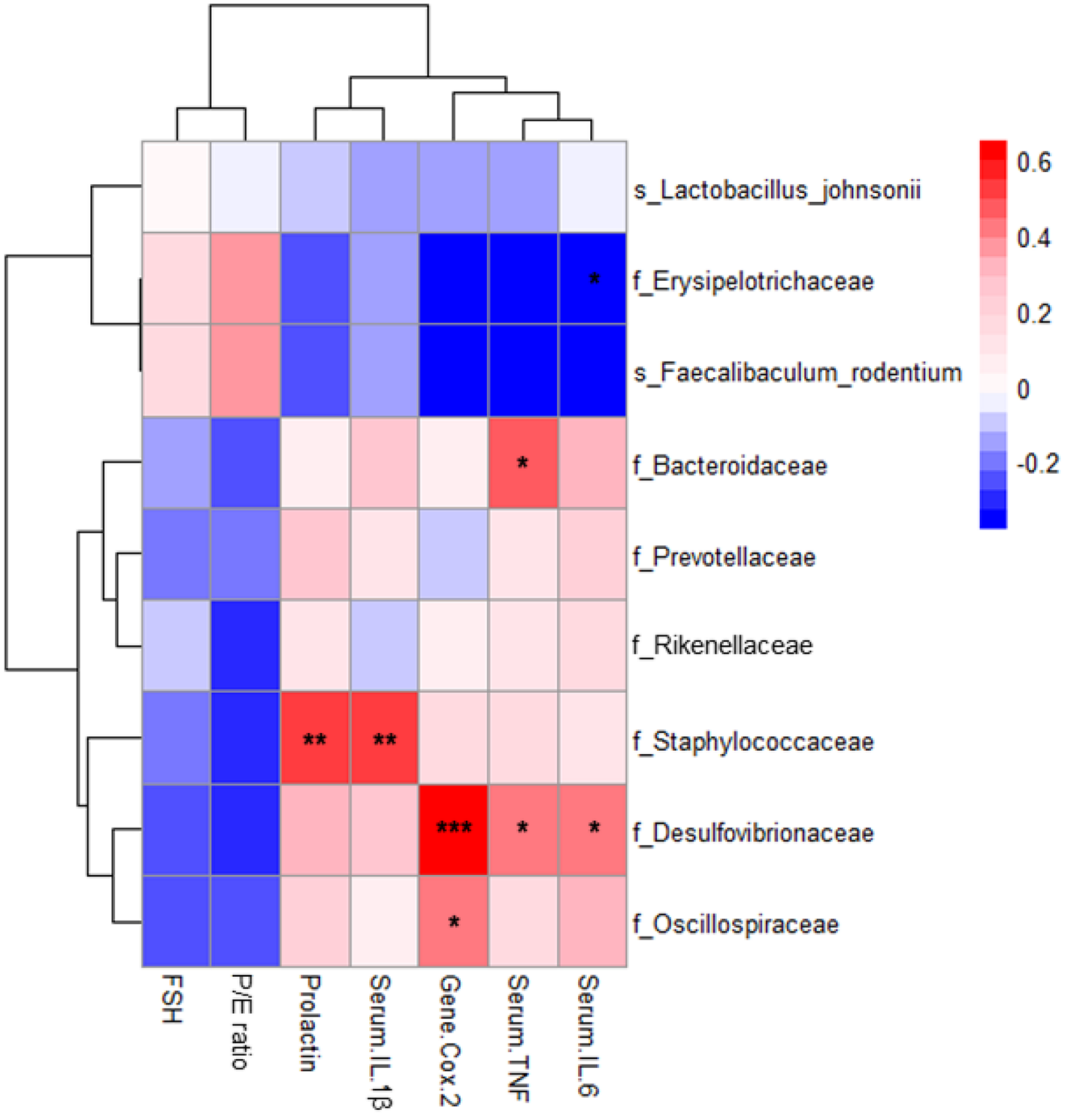
Correlation heatmap between *in vivo* indicators and the composition of the intestinal microbiota (based on Pearson’s correlation coefficient). Red indicates a positive correlation and blue indicates a negative correlation. A *p*-value <0.05 was considered statistically significant. *p < 0.05, **p < 0.01 and ***p < 0.001.

Pearson’s correlation analysis was performed to examine the relationships between microbial abundance and PMS-related biomarkers (Fig. 3). Serum levels of prolactin and pro-inflammatory cytokines (IL-6, TNF, and IL-1β) correlated positively with the increased abundance of *Desulfovibrionaceae*, *Staphylococcaceae*, *Bacteroidaceae*, and *Rinkenellaceae* and negatively correlated with *Erysipelotrichiaceae* and *L. johnsonii*. Expression of *Cox-2* showed a similar correlation pattern. Notably, abundance of *Desulfovibrionaceae* correlated significantly with serum TNF and IL-6 levels (*p* < 0.05), and with and *Cox-2* expression levels (*p* < 0.001). In addition, *Bacteroidaceae* positively correlated significantly with serum TNF levels (*p* < 0.05), and *Staphylococcaceae* positively correlated with levels of prolactin and 1L-1β (*p* < 0.01). By contrast, serum FSH level and Progesteron/Estradiol (P/E) ratio correlated negatively with abundance of *Desulfovibrionaceae*, *Staphylococcaceae* and *Bacteroidaceae* and *Rinkenellaceae*. Additionally, these biomarkers were positively correlated with *Erysipelotrichiaceae*. Taken together, these results suggest that HY7801 may improve PMS-related mental symptoms by modulating the gut microflora and suppressing inflammatory mediators.

### Cytotoxic Effects HY7801-Derived EVs (7801EVs) on GH3 Cells

**Fig. 4:**
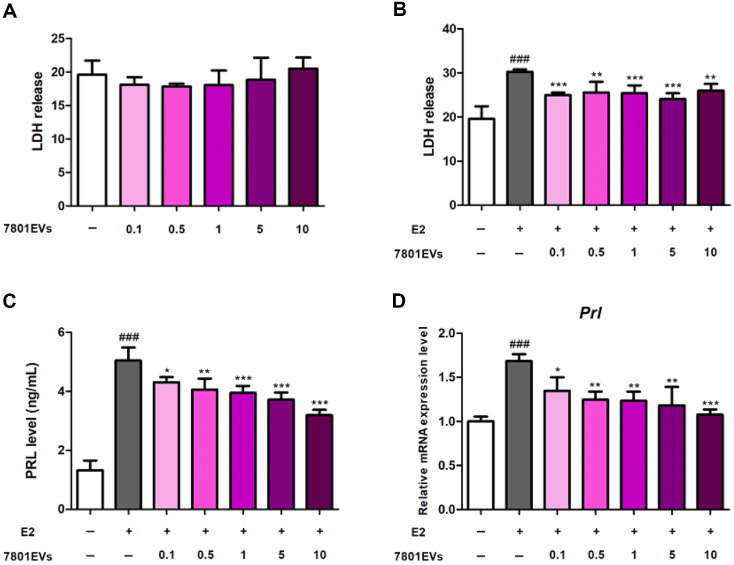
Effect of HY7801-derived EVs on cell cytotoxicity and prolactin levels. (**A**) Cytotoxicity of HY7801-derived EVs. (**B**) Cell cytoprotective effects of HY7801-derived EVs. (**C**) Prolactin secretion levels. (**D**) Expression of Prl. Results are expressed as the mean ± standard deviation. ###*p* < 0.001, compared with untreated cells; *p < 0.05, **p < 0.01 and ***p < 0.001, compared with E2-induced cells. LDH, Lactate Dehydrogenase; PRL, prolactin; 7801EVs, *Lactobacillus helveticus* HY7801- derived extracellular vesicles.

To elucidate the mechanism by which HY7801 improves PMS, *in vitro* tests of functional substances were performed using HY7801-derived EVs (7801EVs) (Fig. S2 and 4). The cytotoxic and protective effects of HY7801-derived EVs on GH3 cells were assessed using the lactate dehydrogenase (LDH) assay. The amounts of LDH released by HY7801-derived EVs-treated cells were comparable with those released by untreated cells, suggestive of no cytotoxicity. As illustrated in Fig. 4B, the LDH assay results revealed that E2 induced a significant increase in LDH release by GH3 cells (*p* < 0.001), suggesting cellular damage. However, treatment with HY7801-derived EVs at concentrations of 0.1, 0.5, 1, 5 or 10 μg/ml attenuated LDH release significantly.

### Effects of HY7801-Derived EVs on Prolactin Secretion by GH3 Cells

To evaluate the effects of HY7801-derived EVs on prolactin, a hormone closely associated with PMS symptoms, prolactin secretion by estradiol (E2)-stimulated GH3 cells was measured (Fig. 4C). Estradiol, a form of estrogen, can accelerate secretion of prolactin by acting on pituitary cells [23]. E2 treatment resulted in prolactin levels of 5.05 ± 0.44 ng/ml, compared with 1.33 ± 0.32 ng/ml in untreated cells (*p* < 0.001). Treatment with HY7801-derived EVs reduced prolactin secretion in a concentration-dependent manner. Specifically, prolactin levels in cells treated with 0.1, 0.5, 1, 5, and 10 μg/ml of EVs were 4.31 ± 0.18 ng/ml (*p* < 0.05), 4.06 ± 0.18 ng/ml (*p* < 0.01), 3.95 ± 0.24 ng/ml (*p* < 0.001), 3.72 ± 0.24 ng/ml (*p* < 0.001), and 3.19 ± 0.18 ng/ml (*p* < 0.001), respectively. Next, we examined whether HY7801-derived EVs affects relative expression of mRNA encoding prolactin (Fig. 4D). Exposure to E2 caused a significant increase in expression of *Prl* (by 1.68-fold compared with the untreated group; *p* < 0.001). Treatment with 0.1, 0.5, 1, 5, or 10 μg/ml HY7801-derived EVs reduced expression of *Prl* significantly and in a concentration-dependent manner (by 1.35- (*p* < 0.05), 1.25- (*p* < 0.01), 1.23- (*p* < 0.01), 1.18- (*p* < 0.01), and 1.10-fold (*p* < 0.001), respectively). Levels of secreted PRL and levels of *Prl* mRNA showed the same pattern, *i.e.*, both fell gradually as the concentration of HY7801-derived EVs increased. Taken together, these results suggest that EVs derived from *L. helveticus* HY7801 may play an important role in suppressing the effects of excessive prolactin secretion, a major cause of PMS.

### Physiological Characteristics of HY7801-Derived EVs

**Fig. 5:**
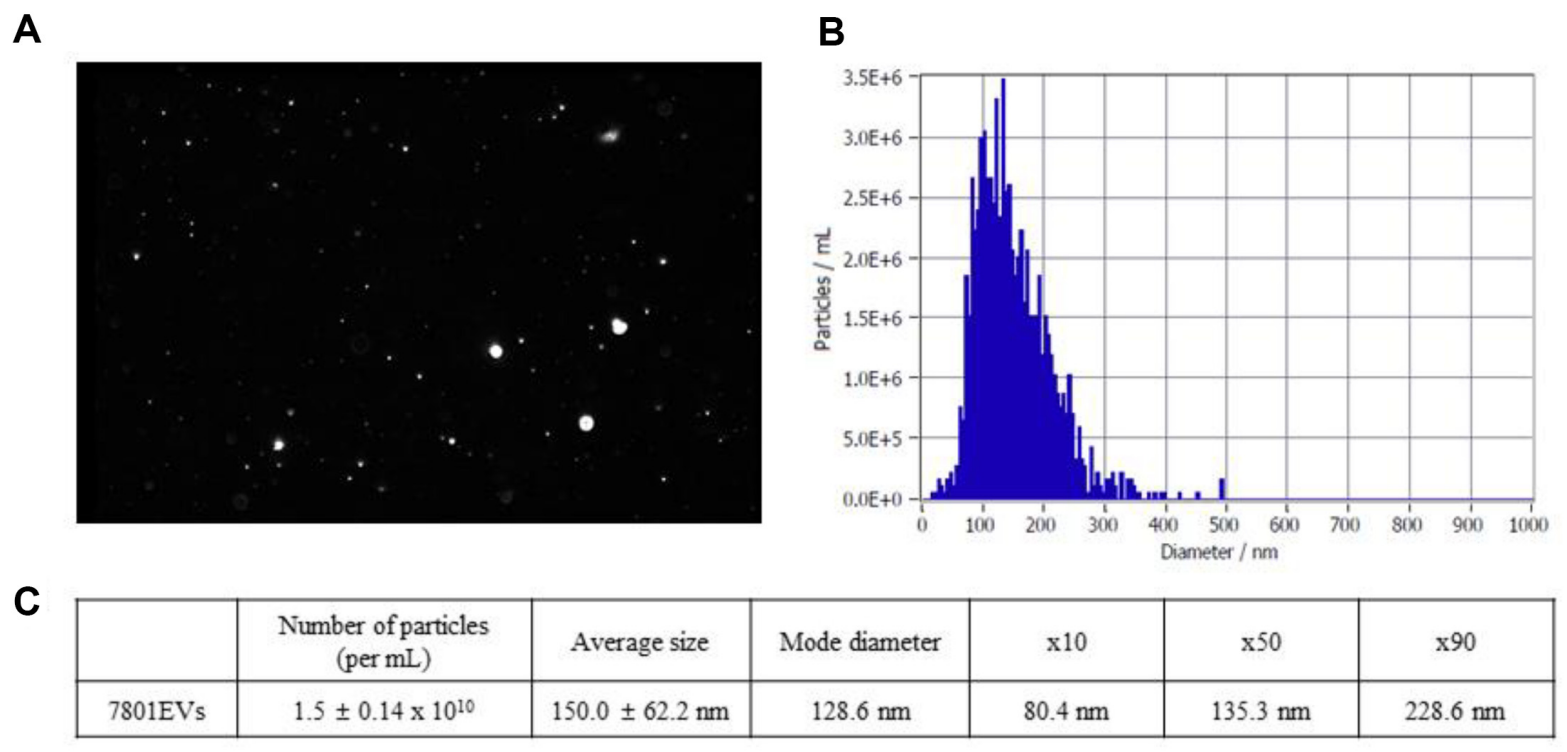
Characteristics and purification of *Lactobacillus helveticus* HY7801-derived extracellular vesicles (7801EVs). (**A**) A representative frame from one of the 7801EV nanoparticle tracking analysis (NTA) videos. (**B**) Size distribution data of 7801EVs, obtain using ZETAVIEW (**C**) The concentration and particle size of 7801EVs was determined by NTA. 7801EVs, *Lactobacillus helveticus* HY7801-derived extracellular vesicles.

Finally, to investigate the characteristics of the EVs from HY7801, NTA was used to analyze the HY7801-derived EVs. A representative image of recorded Brownian motion of nanoparticles in suspension is shown in Fig. 5A and 5B shows the distribution of HY7801-derived EVs based on size. The particle number of EVs per ml was 1.5 ± 0.14 × 10^10^, and the average size was 150.0 ± 62.2 nm (mode diameter: 128.6 nm). The value of X10, X50 and X90 were 80.4, 135.3 and 228.6 nm, respectively (Fig. 5C).

## Discussion

This study aimed to elucidate the mechanisms by which *L. helveticus* HY7801 alleviates symptoms of PMS by examining two primary pathways: modulation of gut the microbiota and the activity of EVs.

We used MCP-induced hyperprolactinemia mice model for PMS study. Currently, there is limitation in that there is a lack of researches on animal models of PMS. MCP is known as medicine which used for stomach and esophageal problems such as vomiting and nausea [24]. In addition, MCP blocks dopamine D2 receptors, which commonly inhibit prolactin secretion [25]. That is, MCP treatment induces prolactin secretion. Abnormal prolactin secretion is one of several hormone disorders suggested as a potential cause of PMS [6]. Previous studies reported that women with PMS symptoms have higher prolactin levels throughout the menstrual cycle, particularly before menstruation [7, 27]. In addition, the resolution of physical and mental symptoms of PMS coincides with a decrease in serum prolactin [28]. Therefore, it appears necessary to reduce the increased level of prolactin in these individuals. In this study, we show that MCP-induced increases in serum prolactin levels, as well as disruption of FSH and the P/E ratio were reversed significantly by HY7801, indicating correction of hormonal dysregulation associated with PMS. These results suggest that administration of HY7801 may alleviate the sex hormone imbalance in the MCP-induced hyperprolactinemia mouse model.

Hyperprolactinemia induces abnormal immune responses [29]. In PMS, inflammation causes physical symptoms such as nonspecific pain in the lower back and abdomen, as well as abdominal bloating [30]. Furthermore, inflammatory mediators and other physiological stimulators increase expression of the *Cox-2* gene, which is closely involved in production of factors that regulate pain [31]. Taken together, our data demonstrate that HY7801 suppresses expression and production of pro-inflammatory cytokines, suggesting that this strain improves abnormal immune responses commonly observed in PMS.

Microbial diversity analysis revealed that MCP increased the α-diversity indices and induced a shift in β-diversity, indicative of dysbiosis. Treatment with HY7801 tended to changemicrobial diversity parameters to levels similar to those observed in the Control group. By contrast, prefemin (PFM) resulted in a pattern of diversity and composition of the microbial flora that was very different from the other groups (Fig. S1). These results suggest that HY7801, but not PFM, can reverse changes to the gut microbiota caused by MCP. Thus, changes in the composition of the intestinal microbiota caused by probiotic strain HY7801 were analyzed. The results showed that the relative abundance of *Desulfovibrionaceae*, *Bacteroidaceae*, *Staphylococcaceae* and *Rinkenellaceae* were reduced by HY7801, while that of beneficial taxa such as *Erysipelotrichaceae* and *Lactobacillus johnsonii* were increased. There was a significant correlation between these microbial taxa and PMS-related biomarkers, including prolactin and inflammatory cytokines. Previous studies showed that individuals with depression may have higher levels of *Desulfovibrionaceae* and lower levels of *Lactobacillus* than healthy individuals [32, 33]. In addition, a reduction in *Desulfovibrionaceae*, *Staphylococcaceae*, and *Bacteroidaceae* and increase in *Erysipelotrichaceae* is associated with mental health conditions such as depression [32, 34, 35]. Furthermore, *Desulfovibrionaceae*, *Staphylococcaceae* and *Rinkenellaceae* are linked to inflammation in the gut [36]. Inflammation is recognized increasingly as a significant factor in development and progression of various mental health conditions, including depression and anxiety [37]. In addition, *Lactobacillus* species are known to play a role in managing stress, thereby potentially preventing mental health conditions such as anxiety and depression [35]. These results indicate that changes the intestinal microbiota (related to mental health and inflammation) are associated with decreased levels of inflammatory cytokines in the MCP-induced mouse model. Taken together, these data support a role for the gut microbiota in modulating the PMS symptoms-alleviating effects of HY7801.

Next, the bioactive functional agents in *L. helveticus* HY7801 were identified. First, the inhibitory effects of live cultures and lysates of HY7801 on secretion of prolactin were examined (Fig. S2). Prolactin levels after exposure to HY7801 live cultures were lower than those after exposure to lysates, with only the results for live cultures being significantly different from those of the E2-induced group (*p* < 0.01). This suggests that metabolic products such as exopolysaccharide (EPS) or EVs produced by the biological activity of HY7801 may have acted as functional agents. EPS, extracellularly secreted polysaccharides produced by *Lactobacillus*, play a crucial role in the probiotic effects because they exhibit antioxidant, anticancer, and anti-inflammatory activities [38]. Extracellular vesicles (EVs) derived from *Lactobacillus* strains are spherical particles ranging from 30 to 200 nm in diameter and are known to carry metabolic intermediates, RNAs, and proteins that can stimulate host immune responses [11, 12]. In this study, HY7801-derived EVs significantly suppressed prolactin secretion in GH3 cells compared with estradiol-stimulated controls (*p* < 0.05). These findings suggest that EVs may serve as key bioactive components responsible for the prolactin-lowering effects of HY7801.

To further clarify the active components in HY7801 EVs, they were used to treat estradiol-stimulated GH3 pituitary cells. LDH is a stable cytosolic enzyme that is released rapidly into the cell culture supernatant when cell membranes are damaged by apoptosis, necrosis, and other forms of cellular assault. Thus, LDH release is a widely used marker for cell cytotoxicity [39]. Our results suggested that HY7801-derived EVs were not cytotoxic; rather, they had a cytoprotective effect on GH3 cells. Additionally, treatment with HY7801-derived EVs suppressed both prolactin secretion and expression of the encoding prolactin (*Prl*) gene in dose-dependent manner, indicating a direct modulatory effect on hormonal pathways associated with PMS. Thus, the data confirmed that HY7801-derived EVs are a bioactive substance responsible for the PMS-alleviating effects of *L. helveticus* HY7801.

Recently, there has been an increasing number of cases in which isolated and purified EVs were used as therapeutic agents. Therefore, to use EVs derived from living cells as therapeutic agents, more information derived from analysis of various characteristics, such as size, size distribution, and concentration, is required [40-42]. NTA revealed that the characteristic of HY7801-derived EVs is consistent with the characteristics of physiologically-relevant EVs. [10-12]. These results suggest that HY7801-derived EVs have properties that could be utilized by the food or pharmaceutical industries as therapeutic agents.

In summary, HY7801 alleviated PMS symptoms by restoring hormonal and immune homeostasis, and by modulating the composition of the gut microbiota. In addition, HY7801-derived EVs were identified as a key bioactive component that regulate prolactin secretion, suggesting their potential as post-biotic agents for PMS management. Nonetheless, further *in vivo* studies are required to confirm the systemic effects of orally-administered HY7801-derived EVs on hormonal regulation and the gut microbiota, and to elucidate how the mechanisms underlying microbe-host interactions affect the pathophysiology of PMS.

## Supplemental Materials

Supplementary data for this paper are available on-line only at http://jmb.or.kr.



## Figures and Tables

**Table 1:** TaqMan probes used to detect gene expression.

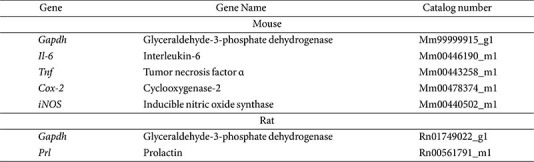

## References

[1] Appleton SM (2018). Premenstrual syndrome: evidence-based evaluation and treatment. Clin. Obstetr. Gynecol..

[2] Ababneh MA, Alkhalil M, Rababa'h A (2023). The prevalence, risk factors and lifestyle patterns of Jordanian females with premenstrual syndrome: a cross-sectional study. Future Sci. OA.

[3] Chumpalova P, Iakimova R, Stoimenova-Popova M, Aptalidis D, Pandova M, Stoyanova M (2020). Prevalence and clinical picture of premenstrual syndrome in females from Bulgaria. Ann. Gen. Psychiatry.

[4] Pullayikudi SPT, Sood A (2024). The clinical impact and management of premenstrual syndrome. Obstet. Gynaecol. Reprod. Med..

[5] Loukzadeh Z, Eslamy N, Dehghan M, Mehrparvar AH (2024). The impact of premenstrual disorders on work disruptions among working women: a cross-sectional study. Int. J. Reprod. BioMed.

[6] Sanchez BN, Kraemer WJ, Maresh CM (2023). Premenstrual syndrome and exercise: a narrative review. Women.

[7] Franchimont P, Dourcy C, Legros J, Reuter A, Vrindts‐Gevaert Y, Cauwenberge JV (1976). Prolactin levels during the menstrual cycle. Clin. Endocrinol..

[8] Abbara A, Clarke SA, Nesbitt A, Ali S, Comninos AN, Hatfield E (2018). Interpretation of serum gonadotropin levels in hyperprolactinaemia. Neuroendocrinology.

[9] Lee YS, Jeon H, Her YM, Lee DE, Jeong YJ, Kim EJ (2021). Lomens-P0 (mixed extracts of *Hordeum vulgare* and *Chrysanthemum zawadskii*) regulate the expression of factors affecting premenstrual syndrome symptoms. Nutr. Res. Practice.

[10] Di Naro M, Petronio Petronio G, Mukhtar F, Cutuli MA, Magnifico I, Falcone M (2025). Extracellular vesicles in bacteria, archaea, and eukaryotes: mechanisms of inter-kingdom communication and clinical implications. Microorganisms.

[11] Zhang Y, Song M, Fan J, Guo X, Tao S (2024). Impact of probiotics-derived extracellular vesicles on livestock gut barrier function. J. Anim. Sci. Biotechnol..

[12] Yang Y, Li N, Gao Y, Xu F, Chen H, Zhang C (2024). The activation impact of *lactobacillus*-derived extracellular vesicles on lipopolysaccharide-induced microglial cell. BMC Microbiol..

[13] Baek J, Lee S, Lee J, Park J, Choi E, Kang S-S (2024). Utilization of probiotic-derived extracellular vesicles as postbiotics and their role in mental health therapeutics. Food Sci. Anim. Resour..

[14] Jo CS, Myung CH, Yoon YC, Ahn BH, Min JW, Seo WS (2022). The effect of *Lactobacillus plantarum* extracellular vesicles from Korean women in their 20s on skin aging. Curr. Issues Mol. Biol..

[15] Sánchez B, Delgado S, Blanco‐Míguez A, Lourenço A, Gueimonde M, Margolles A (2017). Probiotics, gut microbiota, and their influence on host health and disease. Mol. Nutr. Food Res..

[16] Meher AK, Acharya B, Sahu PK (2024). Probiotics: bridging the interplay of a healthy gut and psychoneurological well‐being. Food Bioeng..

[17] Bistas KG, Tabet JP, Bistas K (2023). The benefits of prebiotics and probiotics on mental health. Cureus.

[18] Chelladhurai K, Ayyash M, Turner MS, Kamal-Eldin A (2023). *Lactobacillus helveticus*: health effects, current applications, and future trends in dairy fermentation. Trends Food Sci. Technol..

[19] Kim JY, Moon EC, Kim JY, Kim HJ, Heo K, Shim JJ (2023). *Lactobacillus helveticus* HY7801 ameliorates bacterial vaginosis by inhibiting biofilm formation and epithelial cell adhesion of Gardnerella vaginalis. Food Sci. Biotechnol..

[20] Joo HM, Kim KA, Myoung KS, Ahn YT, Lee JH, Huh CS (2012). *Lactobacillus helveticus* HY7801 ameliorates vulvovaginal candidiasis in mice by inhibiting fungal growth and NF-κB activation. Int. Immunopharmacol..

[21] Kim HJ, Jeong JW, Kim JY, Shim JJ, Lee JH (2024). *Lactobacillus helveticus* HY7801 improves premenstrual syndrome symptoms by regulating sex hormones and inflammatory cytokines in a mouse model of metoclopramide-induced hyperprolactinemia. Nutrients.

[22] Momoeda M, Sasaki H, Tagashira E, Ogishima M, Takano Y, Ochiai K (2014). Efficacy and safety of *Vitex agnus-castus* extract for treatment of premenstrual syndrome in Japanese patients: a prospective, open-label study. Adv. Ther..

[23] Sánchez M, Suárez L, Cantabrana B, Bordallo J (2017). Estradiol-modified prolactin secretion independently of action potentials and Ca2+ and blockade of outward potassium currents in GH 3 cells. Naunyn Schmiedebergs Arch. Pharmacol..

[24] Sanger G (1985). Effects of metoclopramide and domperidone on cholinergically mediated contractions of human isolated stomach muscle. J. Pharm. Pharmacol..

[25] Mccallum RW, Sowers JR, Hershman JM, Sturdevant RA (1976). Metoclopramide stimulates prolactin secretion in man. J. Clin. Endocrinol. Metab..

[26] Graham J, Harding P, Wise P, Berriman H (1978). Prolactin suppression in the treatment of premenstrual syndrome. Med. J. Aust..

[27] Halbreich U, Ben-David M, Assael M, Bornstein R (1976). Serum-prolactin in women witi premenstrual syndrome. Lancet.

[28] Carroll BJ, Steiner M (1978). The psychobiology of premenstrual dysphoria: the role of prolactin. Psychoneuroendocrinology.

[29] Rojas M, Rodríguez Y, Ramírez‐Santana C, Anaya JM (2018). Impact of hyperprolactinemia in a patient with polyautoimmunity. Clin. Case Rep..

[30] Gudipally PR, Sharma GK. Premenstrual syndrome. 2020. 32809533

[31] Simon LS (1999). Role and regulation of cyclooxygenase-2 during inflammation. Am. J. Med..

[32] Liang S, Sin ZY, Yu J, Zhao S, Xi Z, Bruzzone R (2023). Multi-cohort analysis of depression-associated gut bacteria sheds insight on bacterial biomarkers across populations. Cell. Mol. Life Sci..

[33] Johnson D, Thurairajasingam S, Letchumanan V, Chan KG, Lee LH (2021). Exploring the role and potential of probiotics in the field of mental health: major depressive disorder. Nutrients.

[34] Gao J, Pan L, Li P, Liu J, Yang Z, Yang S (2025). Airborne *Staphylococcus aureus* exposure induces depression-like behaviors in mice via abnormal neural oscillation and mitochondrial dysfunction. Environ. Sci. Technol..

[35] Hashikawa-Hobara N, Otsuka A, Okujima C, Hashikawa N (2022). *Lactobacillus paragasseri* OLL2809 improves depression-like behavior and increases beneficial gut microbes in mice. Front. Neurosci..

[36] Maciel-Fiuza MF, Muller GC, Campos DMS, do Socorro Silva Costa P, Peruzzo J, Bonamigo RR (2023). Role of gut microbiota in infectious and inflammatory diseases. Front. Microbiol..

[37] Miller AH, Raison CL (2016). The role of inflammation in depression: from evolutionary imperative to modern treatment target. Nat. Rev. Immunol..

[38] Jurášková D, Ribeiro SC, Silva CC (2022). Exopolysaccharides produced by lactic acid bacteria: from biosynthesis to healthpromoting properties. Foods.

[39] Kumar P, Nagarajan A, Uchil PD (2018). Analysis of cell viability by the lactate dehydrogenase assay. Cold Spring Harb. Protoc..

[40] Grangier A, Branchu J, Volatron J, Piffoux M, Gazeau F, Wilhelm C (2021). Technological advances towards extracellular vesicles mass production. Adv. Drug Deliv. Rev..

[41] Kumar MA, Baba SK, Sadida HQ, Marzooqi SA, Jerobin J, Altemani FH (2024). Extracellular vesicles as tools and targets in therapy for diseases. Signal Transduct. Target. Ther..

[42] Takakura Y, Hanayama R, Akiyoshi K, Futaki S, Hida K, Ichiki T (2024). Quality and safety considerations for therapeutic products based on extracellular vesicles. Pharm. Res..

