# Supplementary material for: A Mechanistic Study of Lactobacillus helveticus HY7801 and Its Extracellular Vesicles in Premenstrual Syndrome: Role of Gut Microbiota and Hormonal Modulation
Source: J Microbiol Biotechnol. 2025 Sep 11;35:e2507014. doi: 10.4014/jmb.2507.07014 (PMC12438957; doi:10.4014/jmb.2507.07014)
Supplement: Supplementary file 1 [file jmb-35-e2507014-supple.pdf]

J. Microbiol. Biotechnol. 2025. 35: e2507014
https://doi.org/10.4014/jmb.2507.07014

Supplementary Figures

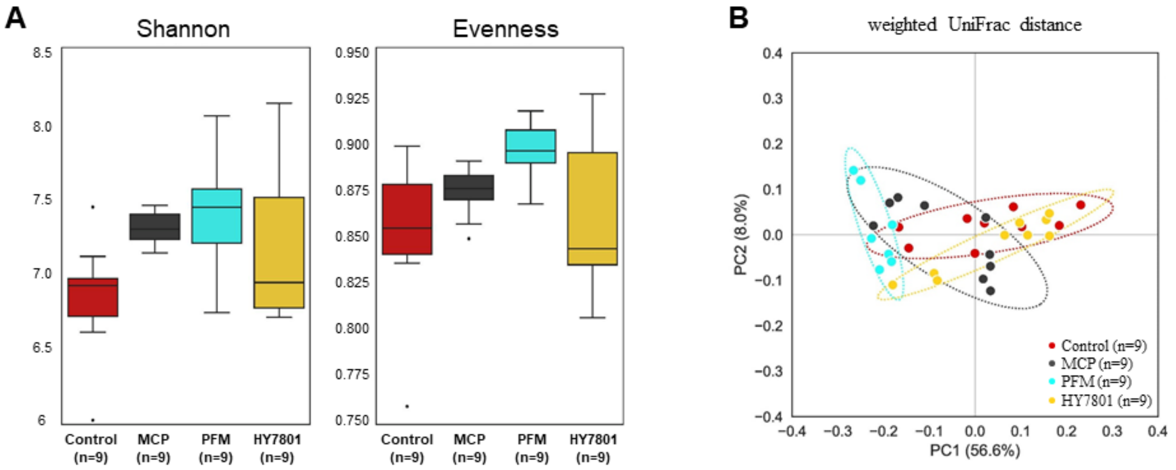

Fig. S1. Diversity of all groups in MCP-induced mouse model. (A) Alpha-diversity, (B) Beta-diversity.

Materials and Methods

Sample Preparation for In vitro tests

Cell lysate of HY7801 was obtained from culture media, and washed twice with sterilized PBS. Resuspended cell lysate in PBS was homogenized using FastPrep-24 (MP Biomedicals, Irvine, CA, USA) and Lysing Matrix B (MP biomedicals) at 6.0 m/s for 30 sec x 5 times.

Exopolysaccharide (EPS) of HY7801 was extracted by ethanol precipitation. Cultured media of HY7801 was centrifuged at 6,500 × g for 30 min at 4 °C. The obtained supernatant was mixed trichloroacetic acid to denature protein, then chilled ethanol was added to the supernatant and incubated for 4 °C at 24 h. The precipitated EPS was resuspended in PBS and dialyzed using a Spectra/Por Dialysis Membrane (Spectrum Laboratories, Inc., USA) with distilled water for 24 h to obtain purified EPS.
Cell lysate and isolated EPS from HY7801 were stored at -80 °C until further in vitro tests.

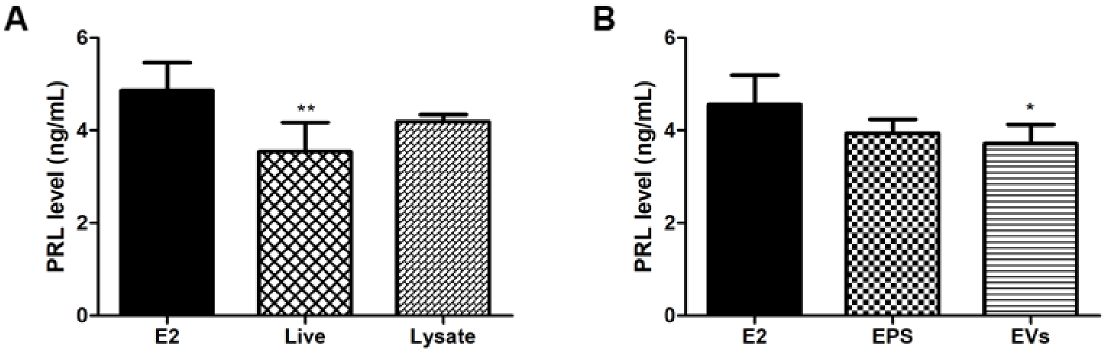

Fig. S2. Prolactin level of HY7801-derived bioactive substances. **(A)** Prolactin level of live culture media and cell lysate, **(B)** Prolactin level of EPS and EVs isolated from HY7801. The results are expressed as the mean ± standard deviation. * $p < 0.05$ and ** $p < 0.01$ compared with E2-induced group.
